# Cohort study on the survival of methotrexate and acitretin treatment in patients with plaque psoriasis: experience from a single center^؆^

**DOI:** 10.1016/j.abd.2025.501225

**Published:** 2025-10-27

**Authors:** Christina de Castro Brommonschenkel, Luciane Donida Bartoli Miot, Maria Cecília Valsechi Belli, Hélio Amante Miot

**Affiliations:** Department of Dermatology, Faculdade de Medicina, Universidade Estadual Paulista, Botucatu, SP, Brazil

Dear Editor,

Psoriasis is an inflammatory, immune-mediated, multifactorial, chronic disease of universal occurrence.^1^ An estimated 125 million individuals are affected worldwide, with five million of them in Brazil alone.^2^, ^3^

Its treatment is based on topical active ingredients for mild cases; however, phototherapy, systemic drugs such as cyclosporine, methotrexate (MTX), acitretin (ACT), immunobiologicals, and small molecules are reserved for moderate and severe forms.^3^

As with other chronic diseases, continuity of therapy is an important aspect in the remission of clinical manifestations. The duration of use of a medication, known as therapeutic survival, is an indicator of its overall effectiveness, reflecting aspects such as adherence, side effects, cost, access, and patient and physician satisfaction.^4^, ^5^

In Brazil, MTX and ACT are first-line therapies for the treatment of moderate to severe psoriasis, available through the Brazilian Unified Health System (SUS, *Sistema Único de Saúde*). However, to date, there are no studies addressing the survival of these drugs in our population or the factors that affect treatment continuity.

With these objectives in mind, a real-world study was conducted with a bidirectional cohort design, based on the analysis of medical records of patients with psoriasis vulgaris who used MTX or ACT as systemic monotherapy at the public psoriasis outpatient clinic of Hospital das Clínicas, FMB-Unesp (Botucatu, SP). The protocol was approved by the institutional ethics committee, and data collection included the analysis of medical records of patients treated between January 2012 and July 2024. Survival curves were estimated using the Kaplan-Meier method, and comparisons between groups were performed using the log-rank test. The effect size was expressed as a Hazard Ratio (HR) with a 95% Confidence Interval.^6^

A total of 274 patients and 346 treatment cycles were evaluated, including 200 MTX treatments and 146 ACT (Table 1). The distribution of ACT treatments showed a prevalence of older age and more elderly patients (≥ 60 years). A higher frequency of patients with arthritis was observed in the MTX group (p < 0.05). ACT was also preferred in the three patients with psoriasis vulgaris associated with pustular palmoplantar psoriasis.

**Table 1:** Demographic and clinical data of the sample of patients with psoriasis vulgaris, with registered visits between January 2012 and July 2024 at the psoriasis outpatient clinic of Hospital das Clínicas of FMB-Unesp (Botucatu, SP), undergoing treatment with methotrexate or acitretin.

Variables	Methotrexate	Acitretin
n	200	146
Female sex, n (%)	97 (48.5)	57 (39.0)
Age, (in years) mean (SD)	55.0 (13.8)	60.2 (14.5)^a^
≥ 60 years, n (%)	87 (43.5)	80 (54.8)^a^
Ethnicity, n (%)		
White	179 (89.5)	128 (87.7)
Brown	15 (7.5)	11 (7.5)
Black	6 (3.0)	6 (4.1)
NA	‒ (‒)	1 (0.7)
Level of schooling, n (%)		
Illiterate	16 (8.0)	12 (8.5)
Elementary School	97 (48.5)	78 (54.9)
High School	51 (25.5)	30 (21.1)
Higher Education	18 (9.0)	14 (9.9)
NA	18 (9.0)	12 (8.2)
Weight, (in kg) mean (SD)	81.2 (17.7)	80.8 (16.9)
≥ 100 kg, n (%)	32 (16.0)	24 (16.4)
Clinical form, n (%)		
Vulgaris	200 (100)	146 (100)
Psoriatic arthritis	54 (27.0)^a^	19 (13.0)
Nail	48 (24.0)	37 (25.3)
Guttate psoriasis	9 (4.5)	7 (4.8)
Inverse psoriasis	10 (5.0)	7 (4.8)
Palmoplantar pustular	‒ (‒)	3 (2.1)^a^
Hyperkeratotic palmoplantar psoriasis	16 (8.0)	20 (13.7)
Scalp	92 (46.0)	58 (39.7)
Others	8 (4.0)	12 (8.2)
Age of onset of psoriasis, (in years) mean (SD)	37.2 (14.9)	40.2 (17.6)
< 30 years, n (%)	60 (30.8)	42 (29.6)
≥ 60 years, n (%)	12 (6.2)	23 (16.2)^a^
Family history of psoriasis, n (%)	24 (12.1)	15 (10.4)
Comorbidities, n (%)		
Arterial hypertension	78 (39.0)	63 (43.2)
Diabetes mellitus	62 (31.0)	39 (26.7)
Depression / Anxiety	46 (23.0)	31 (21.2)
CVD	14 (7.0)	16 (10.9)
Dyslipidemia	86 (43.0)	75 (51.4)

CVD, cardiovascular diseases; NA, Not available.

a p < 0.05 (Methotrexate × Acitretin).

Considering the survival curve (Fig. 1), the estimated median survival for MTX in the sample was 25 (95% CI 22–29) months and for ACT, 32 (95% CI 22–43) months (p = 0.010). The survival rates for MTX were 67%, 32%, and 21%; and ACT was 81%, 39%, and 30% after 1, 3, and 5 years, respectively.

**Fig. 1:**
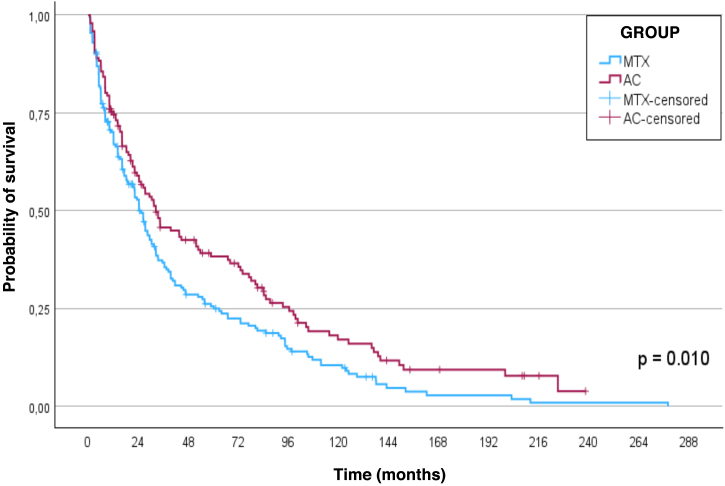
Survival functions (Kaplan–Meier curve) for treatments with methotrexate and acitretin in patients with psoriasis vulgaris treated at the psoriasis outpatient clinic of the Hospital das Clínicas of FMB-Unesp (Botucatu, SP), from January 2012 to July 2024.

Table 2 analyzes survival according to the main covariates. Dyslipidemia was associated with a lower risk of discontinuing treatment with ACT (HR = 0.6) or MTX (HR = 0.5). Late onset of psoriasis (age ≥ 60 years) was associated with a lower risk of discontinuing treatment with MTX (HR = 0.7).

**Table 2:** Hazard Ratio of variables associated with survival of treatments with methotrexate and acitretin in patients with psoriasis vulgaris treated at the psoriasis outpatient clinic of Hospital das Clínicas of FMB-Unesp (Botucatu, SP), from January 2012 to July 2024.

Variables	Methotrexate		Acitretin	
	HR (95% CI)	p-value	HR (95% CI)	p-value
Sex (female)	1.1 (0.8‒1.5)	0.616	0.9 (0.7‒1.4)	0.773
White ethnicity	0.6 (0.4‒1.0)	0.061	0.7 (0.4‒1.4)	0.112
Schooling (Higher Education)	1.4 (0.8‒2.4)	0.213	1.2 (0.6‒2.1)	0.619
Family history	1.1 (0.7‒1.8)	0.645	0.7 (0.4‒1.3)	0.299
Age (≥60 years)	0.7 (0.5‒0.9)	0.015	1.0 (0.7‒1.4)	0.912
Weight (≥80 kg)	1.2 (0.9‒1.6)	0.271	1.1 (0.8‒1.6)	0.615
Age at onset (<30 years)	1.1 (0.8‒1.5)	0.695	0.8 (0.5‒1.1)	0.182
Age at onset (≥60 years)	1.1 (0.5‒2.2)	0.856	1.1 (0.6‒1.9)	0.743
Psoriatic arthritis	1.1 (0.8‒1.6)	0.480	1.5 (0.9‒2.5)	0.087
Nail psoriasis	1.3 (1.0‒1.9)	0.095	0.8 (0.5‒1.3)	0.416
Depression / Anxiety	0.9 (0.6‒1.3)	0.564	1.1 (0.7‒1.7)	0.713
Diabetes mellitus	0.9 (0.7‒1.3)	0.690	1.1 (0.7‒1.7)	0.625
Arterial hypertension	0.8 (0.6‒1.1)	0.102	0.9 (0.6‒1.2)	0.432
Dyslipidemia	0.5 (0.4‒0.7)	<0.001	0.6 (0.4‒0.8)	0.003

HR, Hazard Ratio (95% confidence interval - CI) for treatment discontinuation.

Treatment discontinuation occurred in 86.5% of MTX cases and in 78.8% of ACT cases (Table 3). Treatment failure was the main cause of treatment discontinuation with both MTX (34.5%) and ACT (30.8%).

**Table 3:** Causes of permanent discontinuation of methotrexate or acitretin treatments in patients with psoriasis vulgaris treated at the psoriasis outpatient clinic of the Hospital das Clínicas of FMB-Unesp (Botucatu, SP), from January 2012 to July 2024.

Variables	Methotrexate, n (%)	Acitretin, n (%)
Cases	173 (86.5)	115 (78.8)
Therapeutic failure	69 (34.5)	45 (30.8)
Emergence of contraindication	27 (13.5)	13 (8.9)
Age > 65 years	3 (1.50)	‒
Time of use/Accumulated dose	8 (4.0)	‒
Pregnancy	4 (2.0)	1 (0.7)
Infections	7 (3.5)	1 (0.7)
Comorbidities	6 (3.0)	1 (0.7)
Arthritis/arthralgia	‒	10 (6.8)
Loss to follow-up	28 (14.0)	15 (10.3)
Adverse effect	37 (18.5)	32 (22.0)
Laboratory alteration	15 (7.5)	18 (12.3)
Mucocutaneous symptoms	‒	12 (8.3)
Gastrointestinal symptoms	20 (10.0)	2 (1.4)
Headache	1 (0.5)	‒
Pneumonitis	1 (0.5)	‒
Clinical improvement	18 (9.0)	21 (14.4)
Poor adherence	7 (3.5)	3 (2.1)
Cost	7 (3.5)	‒
Shortage	‒	6 (4.2)
Death	1 (0.5)	1 (0.7)
Others / NA	12 (6.0)	5 (3.4)

NA, Not available.

When comparing treatments initiated before 2020 in relation to those initiated after 2020, the median survivals of patients using MTX were 29 and 12 months (p < 0.01), respectively, whereas in patients who initiated the use of ACT before 2020 and after 2020, the median survival values ​​found were 40 and 19 months (p = 0.025).

The median survival times for MTX and ACT in the population treated at the psoriasis outpatient clinic of Hospital das Clínicas, FMB-Unesp (Botucatu, SP) were slightly higher than the medians reported in the literature (12 to 21 months), demonstrating greater adherence to these treatments in Brazil.^5^, ^6^, ^7^, ^8^, ^9^ This may be explained by the limited availability of more effective treatments, such as immunobiologicals, through the SUS (Unified Health System).

As expected, the main cause of discontinuation of MTX and ACT treatments was treatment failure. In one of the studies, the main reason for discontinuation of MTX treatment was gastrointestinal adverse effects, even with folic acid administration in 99% of patients.^5^ On the other hand, two other studies found that treatment failure was the main cause of discontinuation of MTX treatment.^8^, ^9^ In the dermatology service of FMB-Unesp, it is common to prescribe oral folic acid at a dose of 5 mg/week for patients using MTX, which could help minimize the gastrointestinal effects of MTX, as well as conversion to injectable MTX. Shalom et al. (2015) also observed that the use of oral MTX and folic acid supplementation would be protective factors against the termination of MTX treatment.^7^

The percentage of patients who achieved adherence to MTX and ACT treatments by the end of the third and fifth years of treatment, with a greater superiority in adherence for ACT (30%) compared to MTX (21%) at the end of the fifth year, highlights the importance of these drugs in the long-term treatment of patients with moderate to severe psoriasis in Brazil, where access to new therapies is limited due to availability through the SUS.

Finally, the observation of longer survival rates for both drugs in patients enrolled before 2020 may reflect both the availability of more effective treatments (e.g., anti-IL-23 and anti-IL-17), but also the establishment of more rigorous endpoints for disease control, such as PASI 75-100. Therefore, changes in survival rates also reflect the changing psoriasis treatment flowchart.^3^

A national series showed median survival of less than two years for infliximab and etanercept, while adalimumab showed approximately three years, suggesting that MTX and ACT have survival rates quite comparable to anti-TNF agents.^10^

This study has limitations related to its reliance on medical records, lack of objective metrics (e.g., PASI score), and loss to follow-up, especially during the pandemic.

In conclusion, the median therapeutic survival for methotrexate was 25 months, and for acitretin, 32 months, and the main cause of treatment discontinuation was treatment failure. The data obtained reinforce the role of these drugs in the management of moderate to severe psoriasis in public health services, especially in settings with limited access to advanced therapies.

## ORCID ID

Christina de Castro Brommonschenkel: 0000-0003-2614-9244

Luciane Donida Bartoli Miot: 0000-0002-2388-7842

Maria Cecília Valsechi Belli: 0009-0002-4003-1664

## Financial support

CNPq (306358/2022-0) – Hélio Amante Miot is a CNPq research fellow.

## Authors' contributions

Hélio Amante Miot: Design and planning of the study; analysis and interpretation of data; statistical analysis; drafting and editing of the manuscript; critical review of the literature; critical review of the manuscript; approval of the final version of the manuscript.

Luciane Donida Bartoli Miot: Design and planning of the study; analysis and interpretation of data; drafting and editing of the manuscript; critical review of the literature; critical review of the manuscript; approval of the final version of the manuscript.

Maria Cecília Valsechi Belli: Collection of data; drafting and editing of the manuscript; critical review of the literature; critical review of the manuscript; approval of the final version of the manuscript.

Christina de Castro Brommonschenkel: Design and planning of the study; collection of data; analysis and interpretation of data; statistical analysis; drafting and editing of the manuscript; critical review of the literature; critical review of the manuscript; approval of the final version of the manuscript.

## Availability of research data

The entire dataset supporting the results of this study was published in the article itself.

## Scientific Associate Editor

Luciana P Fernandes Abbade.

## Conflicts of interest

None declared.
